# Validation of microRNA pathway polymorphisms in esophageal adenocarcinoma survival

**DOI:** 10.1002/cam4.989

**Published:** 2017-01-11

**Authors:** Olusola O. Faluyi, Lawson Eng, Xin Qiu, Jiahua Che, Qihuang Zhang, Dangxiao Cheng, Nanjiao Ying, Alvina Tse, Qin Kuang, Lorin Dodbiba, Daniel J. Renouf, Sharon Marsh, Sevtap Savas, Helen J. Mackay, Jennifer J. Knox, Gail E. Darling, Rebecca K. S. Wong, Wei Xu, Abul Kalam Azad, Geoffrey Liu

**Affiliations:** ^1^ Division of Medical Oncology and HematologyDepartment of MedicinePrincess Margaret Cancer Centre and University of TorontoTorontoOntarioCanada; ^2^ Division of Applied Molecular OncologyOntario Cancer Institute‐Princess Margaret Cancer Centre and University of TorontoTorontoOntarioCanada; ^3^ Department of BiostatisticsPrincess Margaret Cancer CentreTorontoOntarioCanada; ^4^ Institute of Biomedical EngineeringHangzhou Dianzi UniversityZhejiangChina; ^5^ British Columbia Cancer AgencyDepartment of Medical OncologyUniversity of British ColumbiaVancouverBritish ColumbiaCanada; ^6^ Faculty of Pharmacy and Pharmaceutical SciencesUniversity of AlbertaEdmontonAlbertaCanada; ^7^ Discipline of GeneticsMemorial University of NewfoundlandSt. John'sNewfoundlandCanada; ^8^ Odette Cancer CentreSunnybrook Health Sciences CentreTorontoOntarioCanada; ^9^ Division of Thoracic SurgeryDepartment of SurgeryToronto General HospitalTorontoOntarioCanada; ^10^ Department of Radiation OncologyPrincess Margaret Cancer CentreTorontoOntarioCanada; ^11^ Dalla Lana School of Public HealthUniversity of TorontoTorontoOntarioCanada; ^12^ Department of Genitourinary Medical OncologyDivision of Cancer MedicineUniversity of Texas MD Anderson Cancer CenterHoustonTexas

**Keywords:** Esophageal adenocarcinoma, miRNA pathways, polymorphisms, prognosis

## Abstract

Polymorphisms in miRNA and miRNA pathway genes have been previously associated with cancer risk and outcome, but have not been studied in esophageal adenocarcinoma outcomes. Here, we evaluate candidate miRNA pathway polymorphisms in esophageal adenocarcinoma prognosis and attempt to validate them in an independent cohort of esophageal adenocarcinoma patients. Among 231 esophageal adenocarcinoma patients of all stages/treatment plans, 38 candidate genetic polymorphisms (17 biogenesis, 9 miRNA targets, 5 pri‐miRNA, 7 pre‐miRNA) were genotyped and analyzed. Cox proportional hazard models adjusted for sociodemographic and clinicopathological covariates helped assess the association of genetic polymorphisms with overall survival (OS) and progression‐free survival (PFS). Significantly associated polymorphisms were then evaluated in an independent cohort of 137 esophageal adenocarcinoma patients. Among the 231 discovery cohort patients, 86% were male, median diagnosis age was 64 years, 34% were metastatic at diagnosis, and median OS and PFS were 20 and 12 months, respectively. *GEMIN3 rs197412* (aHR = 1.37, 95%CI: [1.04–1.80]; *P *=* *0.02), *hsa‐mir‐124‐1 rs531564* (aHR = 0.60, 95% CI: [0.53–0.90]; *P *=* *0.05), and *KIAA0423 rs1053667* (aHR = 0.51, 95% CI: [0.28–0.96]; *P *=* *0.04) were found associated with OS. Furthermore, *GEMIN3 rs197412* (aHR = 1.33, 95% CI: [1.03–1.74]; *P *=* *0.03) and *KRT81 rs3660* (aHR = 1.29, 95% CI: [1.01–1.64]; *P *=* *0.04) were found associated with PFS. Although none of these polymorphisms were significant in the second cohort, *hsa‐mir‐124‐1 rs531564* and *KIAA0423 rs1053667* had trends in the same direction; when both cohorts were combined together, *GEMIN3 rs197412*,* hsa‐mir‐124‐1 rs531564*, and *KIAA0423 rs1053667* remained significantly associated with OS. We demonstrate the association of multiple miRNA pathway polymorphisms with esophageal adenocarcinoma prognosis in a discovery cohort of patients, which did not validate in a separate cohort but had consistent associations in the pooled cohort. Larger studies are required to confirm/validate the prognostic value of these polymorphisms in esophageal adenocarcinoma.

## Introduction

With a five‐fold increased incidence over the past three decades, esophageal adenocarcinoma is one of the most rapidly rising malignancies in the developed world 1. Advances in established treatment regimens including surgery, radiotherapy, and chemotherapy (including targeted therapies such as trastuzumab) have led to modest improvements in survival; approximately 35 months for localized disease, 15 months for locally advanced disease, and 6 months for metastatic disease 2, 3, 4. As one third of the patients with localized disease survive for more than 10 years, this suggests there is heterogeneity among patients 4. Molecular factors which may contribute to this variation are not yet fully understood, but may assist in prognostication and elucidation of therapeutic targets.

MicroRNA (miRNA) molecules are short noncoding RNA molecules that regulate mRNA stability. miRNAs are produced by cleavage of large primary precursors, known as pri‐miRNAs, into pre‐miRNAs. Further modification and subsequent cleavage yields mature miRNAs, which are capable of negatively regulating the expression of genes by binding to the 3′UTRs of the target mRNAs 5. miRNAs regulate/modulate the translation of hundreds of other genes in multiple genetic pathways, have been shown to modulate the transformation of cancer cells and are linked to the etiology, progression, and prognosis of cancer 5, 6, 7. In addition, expression profiles of these miRNA pathway genes have been linked to other (non‐esophageal adenocarcinoma) cancers 5. Specific to esophageal adenocarcinoma, altered tumoral miRNA expression profiles and cell‐free circulating microRNAs have been correlated with prognosis 8, 9.

Although rare, single‐nucleotide polymorphisms (SNPs) in miRNA and miRNA‐processing pathway genes, which may alter the expression, transcription, and processing of miRNA have also been linked with cancer‐related risk and outcomes in a variety of tumor subtypes including esophageal adenocarcinoma risk 5. However to date, the effects of polymorphisms in these pathways on esophageal adenocarcinoma prognosis have not been studied. As SNPs influencing cancer risk may also impact prognostication, analyzing previously identified polymorphisms in miRNA pathways associated with cancer risk in esophageal cancer may help to yield new prognostic biomarkers and possible therapeutic targets for esophageal adenocarcinoma 10.

We performed an evaluation of miRNA and miRNA pathway‐processing genes previously associated with risk of any cancer with esophageal adenocarcinoma prognosis. Our aims of the study are: (1) to identify miRNA and miRNA pathways polymorphisms associated with cancer risk that can serve as prognostic markers of esophageal adenocarcinoma; and (2) to evaluate any previously identified polymorphic prognostic relationships in cancer in a cohort of esophageal adenocarcinoma patients. This information may help to identify new biological pathways that may influence esophageal adenocarcinoma outcomes.

## Materials and Methods

### Study population

The study protocol was approved by the Research Ethics Board of the University Health Network (UHN), Toronto, Ontario, Canada. The study population consisted of patients with a histologically confirmed diagnosis of esophageal adenocarcinoma who were receiving care at Princess Margaret Cancer Centre‐University Health Network (Toronto, Ontario, Canada). Two separate cohorts of patients—(1) a discovery cohort and (2) a validation cohort—were created for this study from a molecular epidemiology study evaluating the association between germline SNPs, esophageal cancer risk, and prognosis. From May 2006 to August 2009, 231 consecutive patients were prospectively enrolled into the discovery cohort. Between August 2009 and January 2013, a second group of 137 consecutive patients were recruited for the validation cohort. The date separating these two datasets was based on the closure of the initial study on August 15, 2009.

### Eligibility criteria

All patients recruited to our study required a histological diagnosis of esophageal or gastro‐esophageal junction adenocarcinoma, were at least 18 years of age at diagnosis, able to communicate in English language, and had no cognitive deficits that would affect ability to consent. The written consent consisted of completing a baseline study questionnaire for epidemiological data, obtaining blood sample collection for genotyping at study entry as well as access to hospital records for regular updates on their clinicopathological data and survival.

### Baseline epidemiological data

The study questionnaire was derived from the Harvard Oncologic Molecular Epidemiological survey 11. This self‐reported questionnaire documented details on sociodemographics, education, occupation, smoking, alcohol consumption, height and weight, weight loss, and coexistent gastrointestinal problems (such as Barrett's esophagus and *Helicobacter pylori* infection) as well as performance status as measured by the patient‐reported Eastern Cooperative Oncology Group (ECOG) score. A positive smoking history was defined as a patient reporting consuming ≥100 cigarettes in their lifetime. Those with a positive smoking history were classified as current or ex‐smokers dependent on their current smoking status at diagnosis. Where relevant, the total number of pack years smoked was obtained through self‐reported number of cigarettes consumed/day and years smoked. Alcohol intake was also documented in terms of standard drinks consumed per week 12.

### Follow‐up, endpoints, and assessment of clinical outcomes

All patients in the discovery cohort were followed up until June 2011, while those in the validation cohort were followed up until July 2014. Follow‐up of the discovery cohort was limited to June 2011 due to research ethics restrictions. The histological diagnosis, location/clinical stage of cancer, treatments received were obtained from the clinical records. For those who underwent surgical resection, successful surgery was defined as R0 margins (> 1 cm). *Curative intent chemotherapy* was defined as that given in the neoadjuvant or adjuvant settings, while radiotherapy was defined as that given with potentially curative intent to the primary tumor; palliative radiation therapy given to metastatic sites were excluded.

We selected two primary endpoints for this study, progression‐free survival (PFS) and overall survival (OS). PFS was defined as the interval between the date of diagnosis and the first date of disease recurrence, progression, or death. OS was defined as the interval between the date of diagnosis and the date of death. For patients lost to regular follow‐up, efforts were made to obtain information on their vital status from cancer registry records. Otherwise, they were censored for either outcome on the date of last follow‐up.

### Candidate polymorphism selection

We performed a comprehensive literature search (NCBI PubMed) on previously published studies assessing SNPs in the miRNA pathway (until 2013). We specifically selected variants that had been assessed in types of esophageal cancer, and further included other variants where there was a putative association with cancer incidence and survival of any cancer type. A list of candidate SNPs was compiled, covering all four areas of the miRNA pathway: including pri‐miRNAs (*let‐7f‐2, mir‐100, mir‐124‐1, mir‐219‐1, mir‐26a‐1, mir‐30‐a, mir‐30‐c, mir‐373*), pre‐miRNAs (mir‐146a, mir‐196a‐2, mir‐492, mir‐499, mir‐604, mir‐608, mir‐631), genes involved in the biogenesis (i.e., cleavage and processing) of miRNAs (*AGO1, AGO2, DGCR8, DICER, DROSHA, GEMIN3, GEMIN4, HIWI, RAN, XPO5*), and genes containing miRNA target sequences (*BMPR1P, CD14orf101, CD86, DAG1, GOLGA7, IL1A, KIAA0423, KRT81, LAMB3, RAN, RYR3, USP9X*) 13, 14, 15, 16, 17, 18, 19, 20. Descriptions of the sequence variants and the respective pathways involved are provided in Table S1.

### Genotyping

Genomic DNA was extracted from peripheral blood lymphocytes using Archive Pure DNA Blood Kits (5 PRIME, Inter Medico, ON, Canada) according to the manufacturer's recommendations. Genotyping was performed using the GoldenGate^®^ Genotyping Assay (Illumina Inc. San Diego, CA) as per the manufacturer's protocol. Briefly, sequence variants were uploaded to Illumina's Assay Design Tool (ADT) (www.illumina.com) for probe design resulting in a custom panel of 384 matrix spots out of which 54 were allocated to miRNA sequence variants. All the sequence variants presenting a functionality score <0.4 and design ability rank <1, which is considered as a lower limit for genotyping success by the manufacturer, were discarded. A total of 5 *μ*L of 50 ng/*μ*L in 10 mmol/L Tris‐HCL pH 8.0, 1 mmol/L EDTA of genomic DNA underwent an allele‐specific oligonucleotide hybridization followed by extension and ligation. A universal polymerase chain reaction (PCR) step for all loci followed with primers labeled with either Cy3 (primer 1) or Cy2 (primer 2). The amplified products were then hybridized to a sentrix array matrix (SAM) and scanned using the Illumina Bead Array Reader (BAR) (Illumina Inc.). The resulting data were analyzed with Beadstudio v.3.0 using the default parameters. Only sequence variants with GenCall scores >0.25 were called and samples were discarded if call rates were below 85%.

Genotyping for significant SNPs identified in the training set was performed using SNaPShot analysis in the validation set. Multiplex PCR was performed in 25 *μ*L of a reaction mixture with a final concentration of each component as: 4 ng/*μ*L of genomic DNA, 0.2 *μ*mol/L of each primer (nine pairs PCR primer mixture), 2.5 mmol/L of MgCl2, 0.2 mmol/L of each dNTP, and 0.04U/*μ*L of Taq polymerase in 1x PCR buffer (KAPA2G Robust PCR Kit). After an initial 2 min denaturation at 95°C, 35 cycles of denaturation at 94°C for 30 sec, annealing at 58°C for 30 sec, and extension at 72°C for 30 sec were followed by a final extension step at 72°C for 5 min in the thermal cycler (GeneAmp9700; Applied Biosystems Foster City, CA). The PCR product (4.0 *μ*L) was incubated at 37°C for 30 min with 2U of Exonuclease I (New England BioLabs) and 2U of shrimp alkaline phosphatase (New England BioLabs). After a 15 min incubation to inactivate the enzyme at 85°C, 1 *μ*L of enzyme‐purified PCR product was mixed with 5 *μ*L of SNaPshot Multiplex Ready Reaction Mix (Applied Biosystems), 1* μ*L of 1 *μ*mol/L nine extension primer mixture, and 3* μ*L of dH2O. This mixture was placed in the thermal cycler and underwent 25 cycles at 96°C for 10 sec, 50°C for 5 sec, and 60°C for 10 sec. When completed, 0.5U of shrimp alkaline phosphatase was added and the reaction mixture was incubated for 60 min at 37°C to stop nonspecific reaction of extension primers to reduce SnaPshot background. Before loading onto the ABI PRISM 310 Genetic Analyzer (Applied Biosystems), 12 *μ*L of HiDi formamide (Applied Biosystems) was added to 1* μ*L of reaction mixture, and samples were heated to 95°C for 5 min. Analyses were performed with GeneScan 3.0 application software (Applied Biosystems). Table S2 illustrates the primers used for initial PCR amplifications and later SNaPshot analysis.

### Statistical analysis

All statistical analyses were conducted on SAS 9.2. Descriptive statistics were used to assess frequencies of sociodemographics and clinicopathological variables for each cohort. For the discovery cohort, univariable analysis using Cox proportional hazard models were used to assess the association of each variable with OS and PFS. Baseline multivariable Cox proportional hazard models for each clinical outcome were created using a backward selection algorithm of all sociodemographic and clinicopathological variable significantly associated with each outcome (*P *<* *0.10) with age also included in the selection algorithm as a clinically important predictor. Adjusted hazard ratios (aHR) were provided with 95% confidence interval (CI). For each genetic polymorphism, the association with each outcome was first assessed using Kaplan–Meier method (log‐rank test). Each polymorphism was then individually added into the baseline multivariable model created for each outcome (OS and PFS) and tested for significance using the Wald Test. We applied the additive model for genetic inheritance in the Cox proportional hazard models to increase the power for screening. Nominal significance level was set as *P *<* *0.05.

For the validation cohort, each SNP identified as significantly associated with OS and PFS in the discovery cohort was evaluated for association with survival using the same multivariable model that had been developed in the training set. As a form of sensitivity analysis in the validation cohort, we also constructed an independent multivariable model using backward selection, as above, for univariable significant predictors associated with OS and/or PFS in the analysis of the validation cohort. In the validation cohort, each SNP associated with OS or PFS in the discovery cohort was reevaluated in this sensitivity model.

In addition, both the discovery and validation cohorts were combined together to assess the genetic associations using both the same multivariable model in the discovery cohort and sensitivity model from the validation cohort.

## Results

### Baseline sociodemographic and clinicopathological characteristics

Baseline sociodemographic and clinical characteristics of our discovery (*n* = 231) and validation (*n* = 137) cohorts can be found in Table 1. The mean and median follow‐up times were 31 and 20 months, respectively, for our discovery cohort and 22 and 17 months for our validation cohort. At the time of analysis, there were 147 (64%) deaths in the discovery cohort and 84 (61%) deaths in the validation cohort. Furthermore, patients with evidence of progressive disease who remained alive were 22 (10%) of the discovery cohort and 19 (14%) of the validation cohort. Median PFS was 12 months for the discovery cohort and 13 months for the validation cohort, while median OS was 20 months for the discovery cohort and 17 months for the validation cohort.

**Table 1:** Summary of patient baseline sociodemographics, clinicopathological, and treatment characteristics of our esophageal adenocarcinoma discovery and validation cohorts

Variable	Subgroup	Discovery Cohort	Validation Cohort	*P* Value
*Sociodemographic variables*				
Gender	Male	86%	85%	0.88
Age at diagnosis	Median (range)	64 (29–88)	62 (29–86)	0.08
Ethnicity	Caucasian	91%	91%	1.00
Occupation	White collar	54%	51%	0.72
Education	Any postsecondary	52%	50%	0.82
Marital status	Married or equivalent	72%	73%	0.80
BMI at diagnosis	Underweight (≤18.5)	4%	2%	0.49
	Overweight (>25)	48%	54%	
Smoking status	Current	14%	29%	0.003
	Ex‐smoker	56%	43%	
Pack years smoked	Median (range)	13.5 (0–118)	15 (0–180)	0.25
Alcohol use	Yes	86%	69%	< 0.001
Years drinking	Median (range)	41 (0–77)	29 (0–70)	< 0.001
*Clinicopathological variables*				
Barrett's esophagus	Yes	19%	15%	0.34
Heart burn	Yes	78%	72%	0.27
*H. pylori*	Yes	4%	4%	1.00
ECOG	0/1+	21%/79%	22%/78%	0.89
Weight loss	Median (range)	5.4 (0–55.9)	5.4 (0–34.4)	0.36
Tumor location	GEJ	40%	39%	0.007
	Distal	50%	43%	
	Middle	8%	2%	
Clinical stage overall	1–3	66%	71%	0.33
	4	34%	29%	
Overall treatment intent	Curative	78%	74%	0.44
Surgery attempted	Yes	69%	59%	0.07
Successful surgery	Yes	62%	53%	0.12
Radiation received	Yes	59%	60%	0.91
Chemotherapy	Adjuvant or NeoAdjuvant	46%	59%	0.02

All values represent percentages of patients except for pack years smoked, years of alcohol drunk, weight loss and age where the median and range in brackets are given. *P* values compare characteristics between the discovery and validation cohorts.

GEJ, gastro‐esophageal junction; ECOG, Eastern Cooperative Oncology Group performance score; BMI, body mass index.

In both cohorts, the majority of patients were male, Caucasian, with a median age in the early 60s, were married, had a smoking history, and experienced heartburn symptoms. Furthermore, the majority had localized tumors, had not experienced a significant amount of weight loss, and were of good performance status. Alcohol consumption was more frequent in the discovery cohort, while ongoing smoking at diagnosis was more prevalent in the validation cohort. Furthermore, a relatively higher proportion of rare mid‐esophageal tumors were observed in the discovery cohort, while curative intent chemotherapy was more commonly given in the validation cohort.

### Quality control of genetic data

Details of the original list of the selected polymorphisms can be found in Supplementary Table 1. The genotype information, MAF and genotypic frequency of the final listing of the 38 polymorphisms investigated in the discovery cohort of the study can be found in Table S2. Two SNPs (*GOLGA7 rs11337*,* MIR30C1 rs16827546*) were excluded due to MAF <5%, two SNPs (*USP9X rs10463*,* hsa‐let‐7f‐2 rs17276588*) were excluded as they are located on the X chromosome where time‐to‐event methodologic approaches have not been developed to take into account X inactivation, and five SNPs (*DGCR8 rs3757*,* DROSHA rs10719*,* hsa‐mir‐100 rs1834306*,* hsa‐mir‐219‐1 rs213210*,* hsa‐mir‐26a‐1 rs7372209*) were excluded for not being in Hardy–Weinberg Equilibrium (*P *<* *0.05).

### Association analysis of polymorphisms and cancer outcomes

Univariable and multivariable analysis of the association between baseline sociodemographic and clinicopathological parameters with OS and PFS is displayed in Table 2. The final multivariable model for OS was adjusted for weight loss, stage, and successful surgery, while the final multivariable model for PFS was adjusted for weight loss, stage, successful surgery, and occupation.

**Table 2:** Univariate and Multivariate Results for our clinical base model for the outcomes of overall survival and progression‐free survival in our discovery cohort

Variable	Comparison	Overall Survival (OS)				Progression‐free Survival (PFS)			
		Unadjusted results		Multivariate results		Unadjusted results		Multivariate results	
		HR (95% CI)	*P* Value	aHR (95% CI)	*P* Value	HR (95% CI)	*P* Value	aHR (95% CI)	*P* Value
Gender	Male vs. female	1.71 (1.00–2.92)	0.05	–	–	1.40 (0.88–2.24)	0.15		
Age At Dx	Per 1 Year increase	1.00 (0.98–1.02)	0.67			1.00 (0.99–1.01)	0.91		
Ethnicity	Caucasian vs. non‐Caucasian	0.80 (0.46–1.39)	0.42			0.87 (0.51–1.49)	0.62		
Occupation	Industry vs. business	1.43 (1.03–2.01)	0.03	–	–	1.43 (1.05–1.95)	0.03	1.47 (1.02–2.10)	0.04
Education	No postsecondary vs. postsecondary	1.21 (0.73–1.70)	0.26			1.25 (0.91–1.72)	0.16		
Marital Status	Married vs. single	1.16 (0.78–1.73)	0.46			1.27 (0.88–1.84)	0.20		
BMI	Overweight vs. normal	1.76 (0.81–3.85)	0.15			2.10 (0.96–4.57)	0.06		
	Underweight vs. normal	0.96 (0.68–1.36)	0.83			1.04 (0.75–1.43)	0.83		
Smoking Status	Ex‐Smoker vs. never	0.81 (0.57–1.15)	0.23			0.81 (0.57–1.15)	0.23		
	Current Smoker vs. never	1.17 (0.74–1.84)	0.50			1.17 (0.74–1.84)	0.51		
Pack Years	Per pack year increase	1.00 (0.99–1.01)	0.42			1.00 (0.99–1.01)	0.57		
Alcohol Use	Yes vs. No	1.34 (0.82–2.19)	0.24			1.34 (0.82–2.20)	0.24		
Years of EtOH	Per year increase	0.99 (0.98–1.01)	0.47			0.99 (0.98–1.00)	0.21		
Barrett's Esophagus	Yes vs. No	0.45 (0.26–0.80)	5.9E‐3	–	–	0.36 (0.21–0.61)	2.0E‐4	–	–
Heart Burn	Yes vs. No	0.97 (0.64–1.46)	0.89			1.05 (0.72–1.54)	0.80		
*H pylori*	Yes vs. No	0.80 (0.35–1.81)	0.59			0.80 (0.35–1.81)	0.59		
ECOG	2–3 vs. 0–1	1.80 (1.05–3.07)	0.03	–	–	1.80 (1.06–3.07)	0.03		
Weight Loss	Per kg lost	1.02 (1.01–1.04)	5.2E‐3	1.02 (1.01–1.04)	5.2E‐3	1.02 (1.01–1.04)	7.0E‐4	1.02 (1.00–1.03)	0.02
Tumor Location	GEJ vs. distal third	0.81 (0.57–1.14)	0.22			0.94 (0.68–1.30)	0.71		
	Middle vs. distal third	1.70 (0.94–3.06)	0.08			1.69 (0.97–2.92)	0.06		
	Upper vs. distal third	0.53 (0.07–3.85)	0.54			0.45 (0.06–3.24)	0.43		
Clinical Stage	4 vs. 1‐3	3.45 (2.45–4.87)	1.6E‐12	1.59 (1.02–2.48)	0.04	3.55 (2.56–4.90)	1.9E‐14	2.10 (1.38–3.21)	6.0E‐4
Treatment Intent	Palliative vs. curative	4.21 (2.92–6.08)	1.5E‐14	–	–	4.61 (3.22–6.64)	1.2E‐16	–	–
Surgery	Successful vs. other	0.25 (0.18–0.34)	1.5E‐16	0.30 (0.19–0.48)	2.1E‐4	0.27 (0.20–0.37)	1.9E‐16	0.40 (0.27–0.61)	2.0E‐5
Radiation	Yes vs. No	1.59 (1.13–2.23)	8.0E‐3	–	–	1.54 (1.12–2.11)	7.8E‐3	–	–
Chemotherapy	Adj/NeoAdj vs. pall/none	0.64 (0.46–0.89)	8.7E‐3	–	–	0.71 (0.53–0.97)	0.03	–	–

Backward selection of clinical variables significantly associated (*P *<* *0.10) with each outcome with age included in the selection algorithm was conducted to create separate significant multivariate clinical base models for each outcome (*P *<* *0.05). It is upon these base models that each genetic polymorphism was then evaluated upon for significance.

Dx, Diagnosis; EtOH, Alcohol; Adj, Adjuvant; NeoAdj, Neoadjuvant; Pall, Palliative; BMI, Body Mass Index.

Univariable and multivariable analysis results of our polymorphisms with OS and PFS for our discovery cohort can be found in Table 3. Univariable analysis identified five polymorphisms that were significantly associated with OS, namely biogenesis pathway gene polymorphisms *GEMIN3 rs197412* and *GEMIN4 rs3744741*, miRNA target gene polymorphisms *CD86 rs17281995* and *KIAA0423 rs1053667*, and pre‐miRNA polymorphism *hsa‐mir‐492 rs2289030*. In multivariable analysis, *GEMIN3 rs197412* and *KIAA0423 rs1053667* remained significantly associated with OS (aHR = 1.37, 95% CI: (1.04–1.80); *P *=* *0.02 and aHR = 0.51, 95% CI: (0.28–0.96); *P *=* *0.04, respectively). In addition, one pri‐miRNA polymorphisms was found to be significantly associated with OS in multivariable analysis that was originally not found associated with OS in univariable analysis—*hsa‐mir‐124‐1 rs531564* (aHR = 0.60, 95% CI: (0.37–0.99); *P *=* *0.05).

**Table 3:** Significant multivariate associations results between the miRNA pathway polymorphisms and prognosis (OS and PFS) in esophageal adenocarcinoma in our discovery cohort

Gene	RS Number	A1	A2	Overall Survival (OS)				Progression‐free Survival (PFS)			
				Unadjusted results		Multivariate results		Unadjusted results		Multivariate results	
				HR (95% CI)	*P* Value	aHR (95% CI)	*P* Value	HR (95% CI)	*P* Value	aHR (95% CI)	*P* Value
GEMIN3	rs197412	G	A	1.34 (1.05–1.72)	0.02	1.37 (1.04–1.80)	0.02	1.31 (1.04–1.66)	0.02	1.33 (1.03–1.74)	0.03
CD86	rs17281995	G	C	1.50 (1.08–2.08)	0.01	1.37 (0.98–1.94)	0.06	1.35 (0.99–1.85)	0.06	1.23 (0.88–1.73)	0.23
hsa**–**mir‐124‐1	rs531564	G	C	0.85 (0.57–1.28)	0.44	0.60 (0.37–0.99)	0.05	0.98 (0.68–1.40)	0.91	0.77 (0.50–1.19)	0.24
hsa‐mir‐492	rs2289030	G	C	0.58 (0.33–1.00)	0.05	0.82 (0.46–1.47)	0.50	0.68 (0.42–1.10)	0.12	0.94 (0.56–1.60)	0.82
KIAA0423	rs1053667	G	A	0.57 (0.32–1.01)	0.05	0.51 (0.28–0.96)	0.04	0.72 (0.44–1.16)	0.17	0.72 (0.42–1.23)	0.22
GEMIN4	rs3744741	A	G	1.39 (1.01–1.91)	0.04	1.25 (0.89–1.79)	0.19	0.72 (0.44–1.16)	0.18	0.72 (0.42–1.23)	0.23
KRT81	rs3660	C	G	1.09 (0.86–1.38)	0.47	1.20 (0.93–1.55)	0.17	1.12 (0.90–1.39)	0.32	1.29 (1.01–1.64)	0.04

The multivariate analysis results were adjusted for weight loss, stage, and surgery for the OS baseline model and adjusted for weight loss, stage, surgery, and occupation for the PFS baseline model.

A1, Minor Allele; A2, Major Allele; aHR are per each risk (minor) allele (A1).

*GEMIN3 rs197412* was found also to be associated with PFS in both univariable analysis and multivariable analysis (aHR = 1.33, 95% CI: (1.03–1.74); *P *=* *0.03). One additional polymorphism was also significantly associated with PFS in multivariable analysis, but not in univariable analysis: *KRT81 rs3660* (aHR = 1.29, 95% CI: (1.01–1.64); *P *=* *0.04).

Upon evaluation of the significant polymorphisms found in multivariable analysis in our validation cohort, none of our identified polymorphisms were found significantly associated with their respective outcomes using either the multivariable modeling from the discovery cohort or with a sensitivity model from backward selection in the validation cohort (Table S3). *KRT81 rs3660* was significantly associated with PFS, but showed opposite directionality (aHR = 0.62, 95% CI: (0.42–0.91); *P *=* *0.02). The strongest identified nonsignificant trend that was consistent in directionality was *hsa‐mir‐124‐1 rs531564* with OS (aHR = 0.72, 95% CI: (0.47–1.11); *P *=* *0.13) (Table 3).

**Figure 1:**
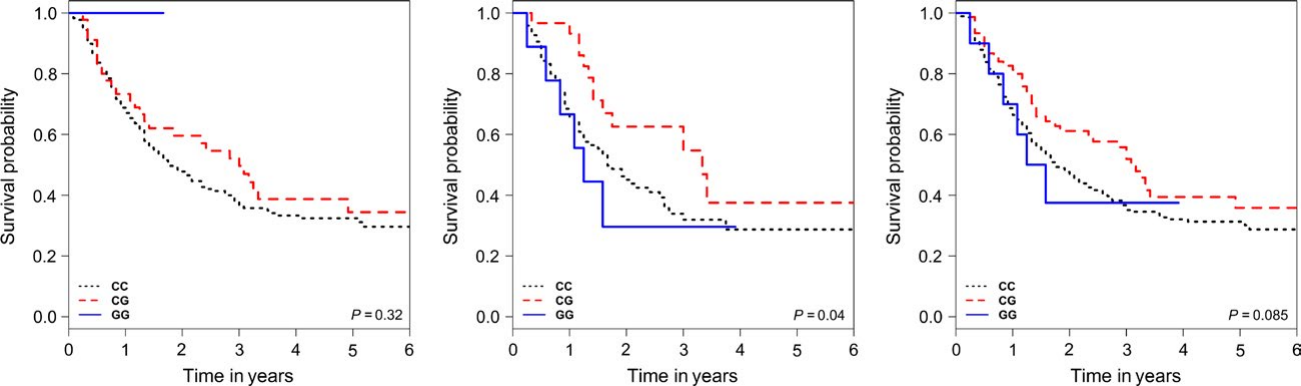
Kaplan–Meier Curves for overall survival with hsa‐mir‐124‐1 *rs531564* in our discovery (left), validation (middle) and combined (right) cohort of patients with esophageal cancer.

Upon combining both the discovery and validation cohorts, *hsa‐mir‐124‐1 rs531564* (aHR = 0.72, 95% CI: (0.52–0.99); *P *=* *0.05) remained significantly associated with OS using the discovery cohort model (Table 4). The Kaplan–Meier curves for *hsa‐mir‐124‐1 rs531564* in the discovery, validation, and combined cohorts can be found in Figure 1. In addition, *KIAA042*3 *rs1053667* was found significantly associated with OS in both the discovery cohort (aHR = 0.56, 95% CI: (0.32–0.97); *P *=* *0.04) and sensitivity analysis model (aHR = 0.64, 95% CI: (0.41–0.99); *P *=* *0.04) (Table 4, Table S4). *GEMIN3 rs197412* was only found significantly associated with OS (aHR = 1.26, 95% CI: (1.03–1.55); *P *=* *0.02) in the sensitivity analysis model (Table 4, Table S4). None of the originally identified polymorphisms were found significantly associated with PFS in the combined cohort.

**Table 4:** Multivariate associations results between the miRNA pathway polymorphisms and overall survival in esophageal adenocarcinoma across all three esophageal adenocarcinoma cohorts (discovery, validation, and combined) among polymorphisms originally found to be significantly associated with overall survival in the discovery cohort

Gene	RS Number	A1	A2	Discovery cohort results		Validation cohort results		Combined cohort results	
				aHR (95% CI)	*P* Value	aHR (95% CI)	*P* Value	aHR (95% CI)	*P* Value
GEMIN3	*rs197412*	C	T	1.37 (1.04–1.80)	0.02	1.05 (0.72–1.54)	0.80	1.19 (0.95–1.49)	0.13
hsa‐mir‐124‐1	*rs531564*	G	C	0.60 (0.37–0.99)	0.05	0.72 (0.47–1.11)	0.13	0.72 (0.52–0.99)	0.045
KIAA0423	*rs1053667*	C	T	0.51 (0.28–0.96)	0.04	0.80 (0.34–1.86)	0.60	0.56 (0.32–0.97)	0.038

The multivariate analysis results were adjusted based upon the original model for the discovery cohort, which included weight loss, stage, and successful surgery.

A1, Minor Allele, A2, Major Allele. aHR are per each risk (minor) allele (A1).

As an exploratory analysis, we evaluated the combined effects of our two most consistently associated SNPs with overall survival (*hsa‐mir‐124‐1 rs531564* and *KIAA0423 rs1053667*) in our combined patient (discovery and validation) cohort. As none of the patients carried more than two risk alleles in total, very few patients either were double heterozygotes (3%) or homozygous for the risk allele (3%), a comparison was done between patients who had at least one risk allele and those with no risk alleles. Patients carrying at least one variant allele were found to have reduced risk of OS (aHR = 0.59, 95% CI: (0.42–0.83); *P *=* *0.002) and reduced risk of PFS (aHR1 = 0.71, 95% CI: (0.51–0.99); *P *=* *0.043).

## Discussion

Despite advances in therapy for esophageal adenocarcinoma, response rates and prognosis both remain poor and the need for new biomarkers and therapeutic targets is imperative. Given the diversity of pathways that are regulated by miRNA, polymorphisms in both miRNA and miRNA‐processing pathway genes may help to identify potential new targets for esophageal adenocarcinoma treatment. Here, by evaluating SNPs in miRNA and miRNA pathway genes previously associated with risk of development of cancer in esophageal adenocarcinoma prognosis, we have identified *hsa‐mir‐124‐1 rs531564* as a relatively consistent predictor of overall survival whereby each variant allele contributed to a 30–40% decrease in mortality. Two additional polymorphisms were identified that may potentially be associated with OS in esophageal adenocarcinoma; namely *KIAA0423 rs1053667* and *GEMIN3 rs197412*.

Previous studies have evaluated for prognostic biomarkers in esophageal adenocarcinoma in a variety of cancer‐related pathways including VEGF/angiogenesis, cell cycle pathways, cell free circulating microRNAs, DNA repair pathways, and a few studies have evaluated the role of SNPs in miRNA pathways in the risk of esophageal cancer and on esophageal squamous cell carcinoma prognosis, but no studies to date have evaluated the role of polymorphisms in miRNA pathway genes on esophageal adenocarcinoma prognosis 9, 21, 22, 23, 24, 25, 26, 27. This is the first study known to date, evaluating the potential for polymorphisms in miRNA pathways as prognostic markers in esophageal adenocarcinoma.

Pri‐mRNA *hsa‐mir‐124‐1 rs531564* is a SNP that has previously been found associated with risk of development of cervical, colorectal, and esophageal squamous cell cancers 28, 29. Bioinformatics analyses have suggested that *rs531564* may modulate the secondary structure of hsa‐mir124‐1 and alter the efficiency of the processing of pri‐miRNA‐124‐1, which can explain the association of different expression levels of mature miRNA‐124 with different alleles of this polymorphism 30. Furthermore, from the RegulomeDB database 31, data suggest that *rs531564* is likely to affect the binding of a transcriptional factor called EZH2, which is involved in chromatin remodeling and gene silencing in cancer, and may alter the aggressiveness of tumors and their progression 32. miRNA‐124 has been described as a tumor suppressor, preventing development of a malignant phenotype in the cancer cell by the downregulation of several pathways including STAT3 signaling in colorectal cancer or EZH2 in gastric and hepatocellular cancers 33, 34, 35. In noncancer studies, mi‐RNA 124 has been suggested to have an immune modulatory role, as it has been found associated with experimental autoimmune encephalomyelitis (EAE), inflammatory bowel disease and hence may modulate the microenvironment by playing an immunosuppressive role to enhance tumorigenesis 36, 37, 38. Furthermore, miRNA‐124 has been found to inhibit ROCK, leading to reduction/modulation in the migration/invasion/aggressiveness of hepatocellular carcinomas, gliomas, and bladder cancer 35, 39, 40. Since the previously demonstrated effects of miRNA‐124 appears to be potentially pro‐tumorigenic in the tumor microenvironment but inhibitory to tumorigenesis in the cancer cell, further studies on the functional effects of the *rs531564* in esophageal adenocarcinoma are necessary. Moreover, when searched in the Haploreg Database, no other SNPs were found to be highly linked with *rs531564* 41. This suggests that this SNP is likely to be the causative locus itself if its prognostic association is established in esophageal adenocarcinoma.

Among cancer studies, *KIAA0423 rs1053667*, a 3′ UTR polymorphism was found not associated with risk or OS in non‐Hodgkin's lymphoma or hepatocellular carcinoma and also not associated with prognosis in multiple myeloma patients undergoing autologous stem cell transplant 19, 42, 43, 44, 45. In addition, in a study evaluating SNP regulation of miRNA expression and colon cancer risk, *rs1053667* was found associated with differential expression of its targeting miRNA, hsa‐miR‐19b‐3p in nontumor colonic tissues, but when comparing tumor versus nontumor tissue, the miRNA showed differential expression while *rs1053667* was found not associated with risk of colon cancer 46. However, given the limited studies on the functional characteristics of *KIAA0423*, further genotype‐to‐phenotype analysis is required to better understand its function in carcinogenesis.

*GEMIN3 rs197412* was also found consistently associated with OS in both the discovery and combined cohorts. *GEMIN3 rs197412* was previously found to be associated with recurrence‐free survival in bladder cancer and overall survival in non‐Hodgkin's lymphoma 47, 48. *GEMIN3 rs197412* was not associated with outcome in hepatocellular carcinoma and studies in colorectal cancer have yielded inconclusive results 49, 50, 51, 52. Genotype‐to‐phenotype analyses are required to better characterize the changes caused by this polymorphism (and the polymorphisms highly linked with them) on its gene product 47.

Landmark clinical trials reported within the past decade which have informed current clinical practice, have demonstrated only modest improvements (6–9%) in OS with peri‐operative chemotherapy for esophageal adenocarcinoma or a 26% improvement in overall survival with trastuzumab therapy for HER‐2‐positive advanced esophageal adenocarcinoma 53, 54. Thus, the need for new biomarkers in the prognostication and treatment of esophageal adenocarcinoma is acute. miRNA has the potential to regulate many cancer‐related pathways ranging from cell proliferation, invasion, and apoptosis (i.e., CDKs, Rb, E2F, and BCL‐2 family genes) and can provide insight into the diagnosis and treatment of cancer 55. Polymorphisms in miRNA can potentially modulate miRNA‐mRNA interaction and potentially create or destroy miRNA binding sites; while those in processing genes can influence the miRNA transcript either through altering transcription, processing, or maturation 5. By studying previously associated polymorphisms associated with either cancer risk or prognosis in other cancers, there is a possibility that the same polymorphisms may be able to predict clinical outcome in esophageal adenocarcinoma, yielding insights into new possible pathways to target for therapeutic agents 10, 56.

There were several limitations to this study. These include the self‐reported nature of the study questionnaire which could be affected by recall and social desirability bias. The relatively early stage of the cancer in most of our patients means that the impact of these polymorphisms on prognosis in advanced stage disease may be missed. Also, given that many of these SNPs identified are linked to other polymorphisms, we cannot ascertain if the biological effects seen are due to these polymorphisms or linked polymorphisms 41. Additionally, we have analyzed a set of polymorphisms previously associated with either cancer risk or prognosis in various cancers and further studies should attempt to identify new polymorphisms for analysis using methods including genome‐wide association studies or tagSNP approaches 23, 57. Furthermore, as this is a single center study analysis and our validation cohort was 50% the discovery cohort size with baseline demographic differences, further validation of this relationship in other esophageal adenocarcinoma cohorts and in other disease sites is warranted. However, this heterogeneity in sociodemographic and clinicopathological variables may help explain the differences seen between the results of our discovery and validation cohorts. Specifically, some of the factors that were different between the cohorts including smoking and alcohol status are known factors that can influence prognosis and may have influenced both the model selection and final results that were obtained 58, 59, 60. However, the heterogeneity in these sociodemographic and clinicopathological variables may support the robustness of the consistent associations between the discovery and combined cohorts that were identified.

In summary, this is the first study to evaluate the prognostic effects of miRNA pathways polymorphisms in a cohort of esophageal adenocarcinoma patients. We have identified multiple polymorphisms in miRNA pathway genes that were found associated with esophageal adenocarcinoma prognosis which was not validated in an independent cohort of esophageal adenocarcinoma patients but was found to have consistent relationships when both cohorts were combined. They were namely: *hsa‐mir‐124‐1 rs531564*,* KIAA0423 rs1053667*, and *GEMIN3 rs197412*. Future studies are needed to validate these identified relationships in other prospective studies of esophageal adenocarcinoma and evaluate their prognostic role in other cancer disease sites.

## Disclaimers

The study sponsors had no role in the design of the study; the collection, analysis, and interpretation of the data; the writing of the manuscript; and the decision to submit the manuscript

## Conflict of Interest

None declared.

## Supporting information


**Table S1.** Summary of candidate polymorphisms belonging to mi‐RNA and mi‐RNA pathways genes selected for inclusion in our study on esophageal adenocarcinoma prognosis.
**Table S2**. Final listing of miRNA pathway polymorphisms investigated and their quality control metrics. A total of 47 polymorphisms were originally selected for investigation in the study and 38 polymorphisms were included in the final analysis. The specific genotype distribution frequency (percentages) is also listed.
**Table S3.** Results of our identified mi‐RNA pathway polymorphisms significantly associated with esophageal adenocarcinoma prognosis (OS and PFS) in our validation cohort.
**Table S4**. Results of the identified mi‐RNA pathway polymorphisms significantly associated with esophageal adenocarcinoma prognosis (OS and PFS) in the combined cohort.
