# Supplementary material for: Validation of microRNA pathway polymorphisms in esophageal adenocarcinoma survival
Source: Cancer Med. 2017 Jan 11;6(2):361–73. doi: 10.1002/cam4.989 (PMC5313634; doi:10.1002/cam4.989)
Supplement: Supplementary file 1 — Table S1. Summary of candidate polymorphisms belonging to mi‐RNA and mi‐RNA pathways genes selected for inclusion in our study on esophageal adenocarcinoma prognosis. Table S2. Final listing of miRNA pathway polymorphisms investigated and their quality control metrics. A total of 47 polymorphisms were originally selected for investigation in the study and 38 polymorphisms were included in the final analysis. The specific genotype distribution frequency (percentages) is also listed. Table S3. Results of our identified mi‐RNA pathway polymorphisms significantly associated with esophageal adenocarcinoma prognosis (OS and PFS) in our validation cohort. Table S4. Results of the identified mi‐RNA pathway polymorphisms significantly associated with esophageal adenocarcinoma prognosis (OS and PFS) in the combined cohort. [file CAM4-6-361-s001.docx]

| **Functional** **Pathway** | **Gene name** | **Literature Name** | **Ref SNPs** | **Location of sequence variant** | **Seq**uence v**ariant** | **Chromosome** | **Reference** |
| --- | --- | --- | --- | --- | --- | --- | --- |
| microRNA biogenesis | AGO1 | Argonaute-1 | rs595961  rs636832 | Intronic  Intronic | A > G  G > A | 1 | Ye et al (12) |
|  | AGO2 | Argonaute-2 | rs4961280 | Promoter | C > A | 8 | Ye et al (12) |
|  | DGCR8 | Di George syndrome critical region gene 8 | rs1640299  rs3757  rs417309 | 3’ UTR  3’ UTR  3’ UTR | C > A  G > A  G > A | 22 | Ye et al (12) |
|  | DICER | Dicer 1, ribonuclease type III | rs13078  rs3742330 | 3’ UTR  3’ UTR | T > A  A > G | 14 | Ye et al (12) |
|  | DROSHA | Drosha | rs10719 | Synonymous coding | G > A | 5 | Yuan et al (19) |
|  | GEMIN3 | Gemin3 | rs197388  rs197412  rs197414 | Upstream  Non-synonymous coding  Non-synonymous coding | T > A  A > G  C > A | 1 | Ye et al (12) |
|  | GEMIN4 | Gemin4 | rs2740348  rs3744741  rs7813  rs910924 | Non-synonymous coding  Non-synonymous coding  Non-synonymous coding  3’ UTR | G > C  G > A  A > G  G > A | 17 | Ye et al (12) |
|  | HIWI | Piwi – like 1 | rs1106042 | Non-synonymous coding | G > A | 12 | Ye et al (12) |
|  | RAN | RAN, member ras oncogene family | rs14035 | 3’ UTR | G > A | 12 | Ye et al (12) |
|  | XPO5 | Exportin 5 | rs11077 | 3’ UTR | A > C | 6 | Ye et al (12) |
| miRNA target | BMPR1P | Bone morphogenetic protein receptor type-1b | rs1434536 | 3’ UTR | A > C | 4 | Saetrom et al (16) |
|  | CD14orf101 | Chromosome 14 open-reading frame 101 | rs4901706 | 3’ UTR | G > A | 14 | Yu et al (18) |
|  | CD86 | T-lymphocyte activation antigen CD86 | rs17281995 | 3’ UTR | C > G | 3 | Landi et al (17) |
|  | DAG1 | Dystrophin-associated glycoprotein-1 | rs12583 | 3’ UTR | G > T | 3 | Yu et al (18) |
|  | GOLGA7 | Golgin A7 | rs11337 | 3’ UTR | G > T | 8 | Yu et al (18) |
|  | IL1A | Interleukin 1-alpha | rs3783553 | 3’ UTR | -/TTCCA | 2 | Gao et al (15) |
|  | KIAA0423  (FAM179B) | Family with sequence similarity 179, member B | rs1053667 | 3’ UTR | T > C | 14 | Yu et al (18) |
|  | KRT81 | Keratin 81 | rs3660 | 3’ UTR | G > C | 12 | Yu et al (18) |
|  | LAMB3 | Laminin-5 β3 | rs2566 | 3’ UTR | G > A | 1 | Zhou et al (14) |
|  | RYR3 | Ryanodine receptor 3 | rs1044129 | 3’ UTR | G > A | 15 | Yu et al (18) |
|  | USP9X | Ubiquitin Specific Peptidase 9, X-Linked | rs10463 | 3’ UTR | A > G | X | Yu et al (18) |
| Pri-miRNA | hsa-let-7f-2 | hsa-let-7f-2 | rs17276588 | Intronic | G > A | X | Ye et al (12) |
|  | hsa-*mir*-100 | hsa-*mir*-100 | rs1834306 | Intergenic | G > A | 11 | Ye et al (12) |
|  | hsa-*mir*-124-1 | hsa-*mir*-124-1 | rs531564 | Intergenic | C > G | 8 | Ye et al (12) |
|  | hsa-*mir*-218 | hsa-*mir*-218 | rs11134527 | Intronic | G > A | 4 | Zhou et al (14) |
|  | hsa-*mir*-219-1 | hsa-*mir*-219-1 | rs107822  rs213210 | Upstream  Upstream | G > A  A > G | 6 | Ye et al (12) |
|  | hsa-*mir*-26a-1 | hsa-*mir*-26a-1 | rs7372209 | Intronic | G > A | 3 | Ye et al (12) |
|  | hsa-*mir*-30a | hsa-*mir*-30a | rs1358379 | Intergenic | A > G | 6 | Ye et al (12) |
|  | hsa-*mir*-30c | hsa-*mir*-30c-1 | rs16827546 | Intronic | C > T | 1 | Ye et al (12) |
|  | hsa-*mir*-373 | hsa-*mir*-373 | rs1298327 | Intergenic | G > A | 19 | Ye et al (12) |
| Pre-miRNA | hsa-*mir*-146a | hsa-*mir*-146a | rs2910164 | Intergenic | C > G | 5 | Ye et al (12) |
|  | hsa-*mir*-196a-2 | hsa-*mir*-196a-2 | rs1161491 | Downstream | G > A | 12 | Ye et al (12) |
|  | hsa-*mir*-492 | hsa-*mir*-492 | rs2289030 | Intergenic | C > G | 12 | Ye et al (12) |
|  | hsa-*mir*-499 | hsa-*mir*-499 | rs3746444 | Intronic | A > G | 20 | Zou et al (13) |
|  | hsa-*mir*-604 | hsa-*mir*-604 | rs2368392 | Intronic | G > A | 10 | Ye et al (12) |
|  | hsa-*mir*-608 | hsa-*mir*-608 | rs4919510 | Intronic | C > G | 10 | Ye et al (12) |
|  | hsa-*mir*-631 | hsa-*mir*-631 | rs5745925 | Intronic | C > G | 15 | Ye et al (12) |

**Supplementary Table 1:** Summary of candidate polymorphisms belonging to mi-RNA and mi-RNA pathways genes selected for inclusion in our study on esophageal adenocarcinoma prognosis.

| **Functional** **Pathway** | **Gene** | **rs Number** | **BP** | **Minor Allele (A1)** | **Major Allele (A2)** | **MAF** | **Genotype Frequencies** | | | **HWE P Value** |
| --- | --- | --- | --- | --- | --- | --- | --- | --- | --- | --- |
|  |  |  |  |  |  |  | **A1/A1** | **A1/A2** | **A2/A2** |  |
| microRNA biogenesis | AGO1 | rs595961 | 36367780 | G | A | 0.20 | 12 (5) | 64 (29) | 147 (66) | 0.20 |
|  | AGO1 | rs636832 | 36363475 | A | G | 0.10 | 2 (1) | 43 (19) | 179 (80) | 1.00 |
|  | AGO2 | rs4961280 | 141647414 | A | C | 0.18 | 6 (3) | 67 (30) | 151 (67) | 0.82 |
|  | DGCR8 | rs1640299 | 20098359 | A | C | 0.45 | 45 (20) | 112 (50) | 66 (30) | 0.89 |
|  | DGCR8 | rs417309 | 20098544 | A | G | 0.06 | 0 (0) | 26 (12) | 198 (88) | 1.00 |
|  | DICER | rs13078 | 95556747 | A | T | 0.21 | 12 (5) | 68 (30) | 144 (64) | 0.31 |
|  | DICER | rs3742330 | 95553362 | G | A | 0.11 | 4 (2) | 41 (18) | 179 (80) | 0.31 |
|  | GEMIN3 | rs197388 | 112297482 | A | T | 0.15 | 3 (1) | 60 (27) | 161 (72) | 0.43 |
|  | GEMIN3 | rs197412 | 112308953 | G | A | 0.34 | 22 (10) | 110 (49) | 92 (41) | 0.24 |
|  | GEMIN3 | rs197414 | 112309123 | A | C | 0.11 | 2 (1) | 45 (20) | 177 (79) | 1.00 |
|  | GEMIN4 | rs2740348 | 649935 | C | G | 0.17 | 7 (3) | 60 (27) | 157 (70) | 0.63 |
|  | GEMIN4 | rs3744741 | 649232 | A | G | 0.15 | 5 (2) | 56 (25) | 163 (73) | 1.00 |
|  | GEMIN4 | rs7813 | 648186 | G | A | 0.44 | 41 (18) | 114 (51) | 69 (31) | 0.68 |
|  | GEMIN4 | rs910924 | 655920 | A | G | 0.27 | 15 (7) | 93 (42) | 116 (52) | 0.62 |
|  | HIWI | rs1106042 | 130841638 | A | G | 0.06 | 1 (0) | 25 (11) | 198 (88) | 0.57 |
|  | RAN | rs14035 | 131361241 | A | G | 0.31 | 22 (10) | 95 (42) | 107 (48) | 0.88 |
|  | XPO5 | rs11077 | 43490947 | C | A | 0.39 | 38 (17) | 98 (44) | 88 (39) | 0.26 |
| miRNA target | BMPR1P | rs1434536 | 96075965 | G | A | 0.50 | 54 (24) | 115 (52) | 54 (24) | 0.69 |
|  | CD86 | rs17281995 | 121839641 | G | C | 0.14 | 4 (2) | 55 (25) | 165 (74) | 1.00 |
|  | CD14orf101 | rs4901706 | 57114385 | A | G | 0.08 | 2 (1) | 31 (14) | 191 (85) | 0.63 |
|  | DAG1 | rs12583 | 49571462 | A | C | 0.25 | 18 (8) | 76 (34) | 130 (58) | 0.16 |
|  | IL1A | rs3783553 | 113531715 | A | T | 0.31 | 24 (11) | 91 (41) | 109 (49) | 0.44 |
|  | KIAA0423 (FAM179B) | rs1053667 | 45543038 | G | A | 0.06 | 0 (0) | 28 (13) | 195 (87) | 1.00 |
|  | KRT81 | rs3660 | 52679937 | C | G | 0.49 | 53 (24) | 115 (51) | 56 (25) | 0.79 |
|  | LAMB3 | rs2566 | 209788514 | A | G | 0.26 | 14 (6) | 88 (39) | 122 (54) | 0.86 |
|  | RYR3 | rs1044129 | 34158266 | A | G | 0.28 | 18 (8) | 89 (40) | 115 (52) | 0.87 |
| Pri-miRNA | hsa-*mir*-124-1 | rs531564 | 9760699 | G | C | 0.11 | 1 (0) | 45 (20) | 177 (79) | 0.48 |
|  | hsa-*mir*-218 | rs11134527 | 168195356 | A | G | 0.25 | 15 (7) | 81 (36) | 128 (57) | 0.72 |
|  | hsa-*mir*-219-1 | rs107822 | 33175575 | A | G | 0.28 | 20 (9) | 87 (39) | 117 (52) | 0.51 |
|  | hsa-*mir*-30a | rs1358379 | 72113442 | G | A | 0.06 | 2 (1) | 23 (10) | 197 (89) | 0.18 |
|  | hsa-*mir*-373 | rs12983273 | 54291832 | A | G | 0.15 | 6 (3) | 56 (25) | 162 (72) | 0.61 |
| Pre-miRNA | hsa-*mir*-146a | rs2910164 | 159912418 | G | C | 0.25 | 17 (8) | 77 (34) | 130 (58) | 0.28 |
|  | hsa-*mir*-196a-2 | rs11614913 | 54385599 | A | G | 0.38 | 27 (12) | 115 (51) | 82 (37) | 0.20 |
|  | hsa-*mir*-492 | rs2289030 | 95228286 | G | C | 0.07 | 0 (0) | 32 (14) | 192 (86) | 0.61 |
|  | hsa-*mir*-499 | rs3746444 | 33578251 | G | A | 0.20 | 10 (4) | 68 (30) | 145 (65) | 0.53 |
|  | hsa-*mir*-604 | rs2368392 | 29834003 | A | G | 0.27 | 21 (9) | 78 (35) | 124 (56) | 0.12 |
|  | hsa-*mir*-608 | rs4919510 | 102734778 | G | C | 0.20 | 13 (6) | 65 (29) | 146 (65) | 0.15 |
|  | hsa-*mir*-631 | rs5745925 | 75645967 | G | C | 0.07 | 2 (1) | 28 (13) | 194 (87) | 0.31 |

Supplementary Table 2. Final listing of miRNA pathway polymorphisms investigated and their quality control metrics. A total of 47 polymorphisms were originally selected for investigation in the study and 38 polymorphisms were included in the final analysis. The specific genotype distribution frequency (percentages) are also listed. Among the 9 polymorphisms excluded from the final listing were *USP9X* rs10463, *hsa-let-7f-2* rs17276588 for being on the X chromosome, *GOLGA7* rs11337*, MIR30C1 rs16827546* for MAF < 5% and *DGCR8* rs3757, *DROSHA* rs10719, *hsa-mir-100* rs1834306, *hsa-mir-219-1* rs213210 and *hsa-mir-26a-1* rs7372209 for not being in Hardy-Weinberg Equilibrium (HWE) (P < 0.05). A1 = minor allele, A2 = major allele.

| Gene | RS Number | A1 | A2 | **Overall Survival (OS)** | | | | **Progression Free Survival (PFS)** | | | |
| --- | --- | --- | --- | --- | --- | --- | --- | --- | --- | --- | --- |
|  |  |  |  | **Discovery Cohort Model** | | **Sensitivity Analysis Model** | | **Discovery Cohort Model** | | **Sensitivity Analysis Model** | |
|  |  |  |  | aHR (95% CI) | P Value | aHR (95% CI) | P Value | aHR (95% CI) | P Value | aHR (95% CI) | P Value |
| GEMIN3 | rs197412 | C | T | 1.05 (0.72-1.54) | 0.80 | 0.99 (0.69-1.40) | 0.40 | 1.15 (0.81-1.64) | 0.44 | 1.09 (0.81-1.45) | 0.57 |
| hsa-mir-124-1 | rs531564 | G | C | **0.72 (0.47-1.11)** | **0.13** | 0.79 (0.52-1.22) | 0.29 | 0.94 (0.62-1.42) | 0.76 | 0.91 (0.64-1.29) | 0.58 |
| KIAA0423 | rs1053667 | C | T | 0.80 (0.34-1.86) | 0.60 | 0.93 (0.47-1.86) | 0.85 | 0.72 (0.34-1.54) | 0.40 | 1.05 (0.60-1.85) | 0.86 |
| KRT81 | rs3660 | C | G | 0.91 (0.61-1.34) | 0.62 | 0.92 (0.65-1.30) | 0.63 | **0.62 (0.42-0.91)** | **0.02** | **0.73 (0.54-1.00)** | **0.05** |

**Supplementary Table 3.** Results of our identified mi-RNA pathway polymorphisms significantly associated with esophageal adenocarcinoma prognosis (OS and PFS) in our validation cohort. The association of these polymorphisms were evaluated upon two separate multivariate models: 1) the discovery cohort model for each outcome (see Table 2) and 2) a sensitivity analysis model which adjusted for an independent backward selection model created for each outcome using significant socio-demographic and clinico-pathological factors associated with each outcome in our validation cohort. The final models for our sensitivity analysis model include treatment intent, receiving surgery and ECOG for OS and treatment intent and receiving surgery for PFS. A1 = Minor Allele, A2 = Major Allele. aHR are per each risk (minor) allele (A1).

| Gene | RS Number | A1 | A2 | **Overall Survival (OS)** | | | | **Progression Free Survival (PFS)** | | | |
| --- | --- | --- | --- | --- | --- | --- | --- | --- | --- | --- | --- |
|  |  |  |  | **Discovery Cohort Model** | | **Sensitivity Analysis Model** | | **Discovery Cohort Model** | | **Sensitivity Analysis Model** | |
|  |  |  |  | aHR (95% CI) | P Value | aHR (95% CI) | P Value | aHR (95% CI) | P Value | aHR (95% CI) | P Value |
| GEMIN3 | rs197412 | C | T | 1.19 (0.95-1.49) | 0.13 | 1.26 (1.03-1.55) | **0.023** | 1.13 (0.90-1.41) | 0.30 | 1.16 (0.97-1.40) | 0.11 |
| hsa-mir-124-1 | rs531564 | G | C | 0.72 (0.52-0.99) | **0.045** | 0.88 (0.66-1.18) | 0.39 | 0.80 (0.58-1.09) | 0.16 | 0.97 (0.76-1.23) | 0.79 |
| KIAA0423 | rs1053667 | C | T | 0.56 (0.32-0.97) | **0.038** | 0.64 (0.41-0.99) | **0.044** | 0.77 (0.48-1.23) | 0.27 | 0.81 (0.56-1.16) | 0.25 |
| KRT81 | rs3660 | C | G | 1.11 (0.89-1.39) | 0.35 | 0.98 (0.81-1.19) | 0.84 | 1.04 (0.84-1.30) | 0.69 | 0.96 (0.80-1.14) | 0.61 |

**Supplementary Table 4**. Results of the identified mi-RNA pathway polymorphisms significantly associated with esophageal adenocarcinoma prognosis (OS and PFS) in the combined cohort. The association of these polymorphisms were evaluated upon two separate multivariate models: 1) the discovery cohort model for each outcome and 2) a sensitivity analysis model. A1 = Minor Allele, A2 = Major Allele. aHR are per each risk (minor) allele (A1).
